# A randomized, double-blind, placebo-controlled trial on the efficacy of ginger in the prevention of abdominal distention in post cesarean section patients

**DOI:** 10.1038/s41598-018-25200-6

**Published:** 2018-05-01

**Authors:** Wasinee Tianthong, Vorapong Phupong

**Affiliations:** Department of Obstetrics and Gynecology, Faculty of Medicine, Chulalongkorn University, Pathumwan, Bangkok, 10330 Thailand

## Abstract

The objective of the study was to evaluate the efficacy of ginger in the prevention of abdominal distention in post cesarean section patients. A randomized, double-blind, placebo controlled trial was conducted. One hundred and seventy-eight post cesarean section patients were either randomized to the study group receiving oral ginger capsules or to the placebo group receiving oral placebo capsules. The average age of the studied women was 32.3 years. The incidence of postoperative abdominal distention was not different between the ginger and the placebo groups (20.2% vs 29.2%, p = 0.328). The efficacy to relieve abdominal distention was superior in the ginger group than the placebo group (91% vs 65.2%, p < 0.001). With regards to quality of life, the number of patients who had the ability to eat was higher in the ginger group than in the placebo group (59.6% vs 43.8%, p = 0.035). There were no significant differences in time to first flatus, maternal satisfaction, and side effects. Ginger does not decrease the incidence of post cesarean section abdominal distention. But, ginger is more effective than the placebo in relieving the severity of abdominal distention on the fourth day after operation and improving the ability to eat.

## Introduction

Cesarean delivery is defined as the birth of a fetus through incisions in the abdominal wall and the uterine wall. Nowadays, there are more cesarean deliveries. The cesarean delivery rate in the United States rose from 4.5% of all deliveries in 1970 to 32.8% in 2010^1^. After cesarean section, most patients will experience some degree of postoperative ileus which is a delay in gastrointestinal motility that occurs after abdominal surgery. The symptoms of postoperative ileus include nausea, vomiting, abdominal distention, abdominal tenderness, and delayed passage of flatus and stool^2^. Previous studies report that abdominal distention occurred in 20–40% of post cesarean patients^3^.

Post cesarean section abdominal distention may not be a serious complication but occurs frequently. It makes patients uncomfortable and affects their quality of life. Currently, there is no agent that proved to be effective in preventing this symptom^2^. Aside from early ambulation, antiflatulent and laxative drugs, some patients try to use alternative medicine such as ginger to relieve their abdominal distention^4^.

Ginger has a long history of medicinal use to relieve abdominal discomfort, nausea and vomiting because it has anti-inflammatory and antioxidant effect^5,6^. It can stimulate digestion, absorption, relieve constipation and flatulence by increasing gastrointestinal motility^7,8^. Hu *et al*. found that ginger can stimulate gastric emptying^9^. There have been studies that have demonstrated that ginger can reduce nausea, vomiting during pregnancy^10,11^, reduce chemotherapy induced nausea and vomiting^11,12^, reduce incidence of nausea and vomiting after cesarean section^13^, and can prevent postoperative nausea and vomiting^6^. Combined of ginger and artichoke extract have an efficacy on the treatment of functional dyspepsia^14^. Combined of ginger and echinacea extract also have an efficacy on inflammation and chronic pain in knee arthrosis^15^.

To date, there has been no clinical trial to investigate the use of ginger in the prevention of abdominal distention in post cesarean section women. Thus, the primary aim of this study was to evaluate the efficacy of ginger in the prevention of abdominal distention in post cesarean section women. Secondary aims were to evaluate the severity of abdominal distention, quality of life, time to first flatus and defecation, maternal satisfaction, and side effects.

## Subjects and Methods

This randomized, double-blinded, placebo-controlled trial was performed at the Department of Obstetrics and Gynecology, Faculty of Medicine, Chulalongkorn University, Bangkok, Thailand, between June 2016 and June 2017. This study was approved by the Research Ethics Committee of the Faculty of Medicine, Chulalongkorn University. The methods were performed in accordance with approved guidelines. Written informed consent was obtained from all participants. This clinical trial was registered at ClinicalTrials.gov (Clinical trials registration: NCT02809027; registered on June 16, 2016).

Post cesarean section women aged 18 to 40 years within 24 hours postpartum were invited to join this study. Women with operative time more than 1 hour, had another procedure during cesarean section such as appendectomy or ovarian cystectomy, already had abdominal distention, had a history of carminative drugs use, and known allergy to ginger were excluded.

After the study was approved, eligible women who gave informed consent were enrolled. All women were cared with the same postoperative protocols which antiflatulent and laxative drugs were not routinely prescribed. They were allowed to drink water within 24 hours after operation. Regular diet was allowed at 48 hours post operation.

The participants were randomized into two groups: treatment or placebo groups. A randomization scheme was generated by random number table using a block-of-four technique. The co-investigator, who had no contact with the patients, generated the allocation sequence prior to the study. The nurses enrolled and assigned the participants to their respective groups. The drugs and placebo were prepared prior to the study by a pharmacist who was not involved in the study. 500 mg of ginger was put into a capsule and no drug was placed in the placebo capsule. As soon as a study subject met the inclusion criteria, the nurses proceeded to select a sequentially numbered opaque envelope.

The opaque envelopes were sequentially labeled and contained 18 capsules of ginger or placebo (identical in size, shape and color). To ensure randomization, each envelope was distributed in a sequential numerical order. Both health care providers and study participants were masked to treatment assignment. Ginger (Zingiber officinale Roscoe, 500 mg per capsule) (Abhaibhubejhr, Thailand) was assigned to the treatment group and corresponding placebo to the placebo group. Drug dose was 2 capsules three times after meal. Treatment was started when the women started drinking water and continued for 3 days. Treatment assignment was not revealed until data collection was completed. All women were admitted into the postpartum ward and discharged on the fourth day after operation. Thus, all women took all of their medications. All women completed the diary chart and returned it at the end of the study.

The primary outcome was the incidence of abdominal distention after operation. Secondary outcomes were to assess the severity of abdominal distention measured by a 100-mm visual analogue scale at first- fourth day after operation, quality of life (in the aspects of eating, sleeping, daily activity, and emotion) at fourth day after operation, time to first flatus and defecation, maternal satisfaction, and side effects. Satisfaction answer choices consisted of the following: very satisfied, satisfied, neutral, unsatisfied, and very unsatisfied.

The sample size calculation was based upon the incidence of abdominal distention. The rate of abdominal distention in post cesarean section patients from a pilot study was 40%. We expected a 50% decrease rate of abdominal distention. With adjustments for a withdrawal rate of 10%, a minimum of 89 women in each group were required to detect statistical difference (α = 0.05, β = 0.2). Thus, a total of 178 women were required for this study.

SPSS version 22 (SPSS Inc, Chicago, IL, USA) was used for statistical analysis. Chi-square test was used for categorical variables. Independent t-test was used for continuous variables. Mann-Whitney U test was used for nonparametric variables when appropriate. A p < 0.05 was considered statistically significant. Analysis of the trial was conducted by using intent-to-treat (ITT) analysis.

## Results

**Figure 1:**
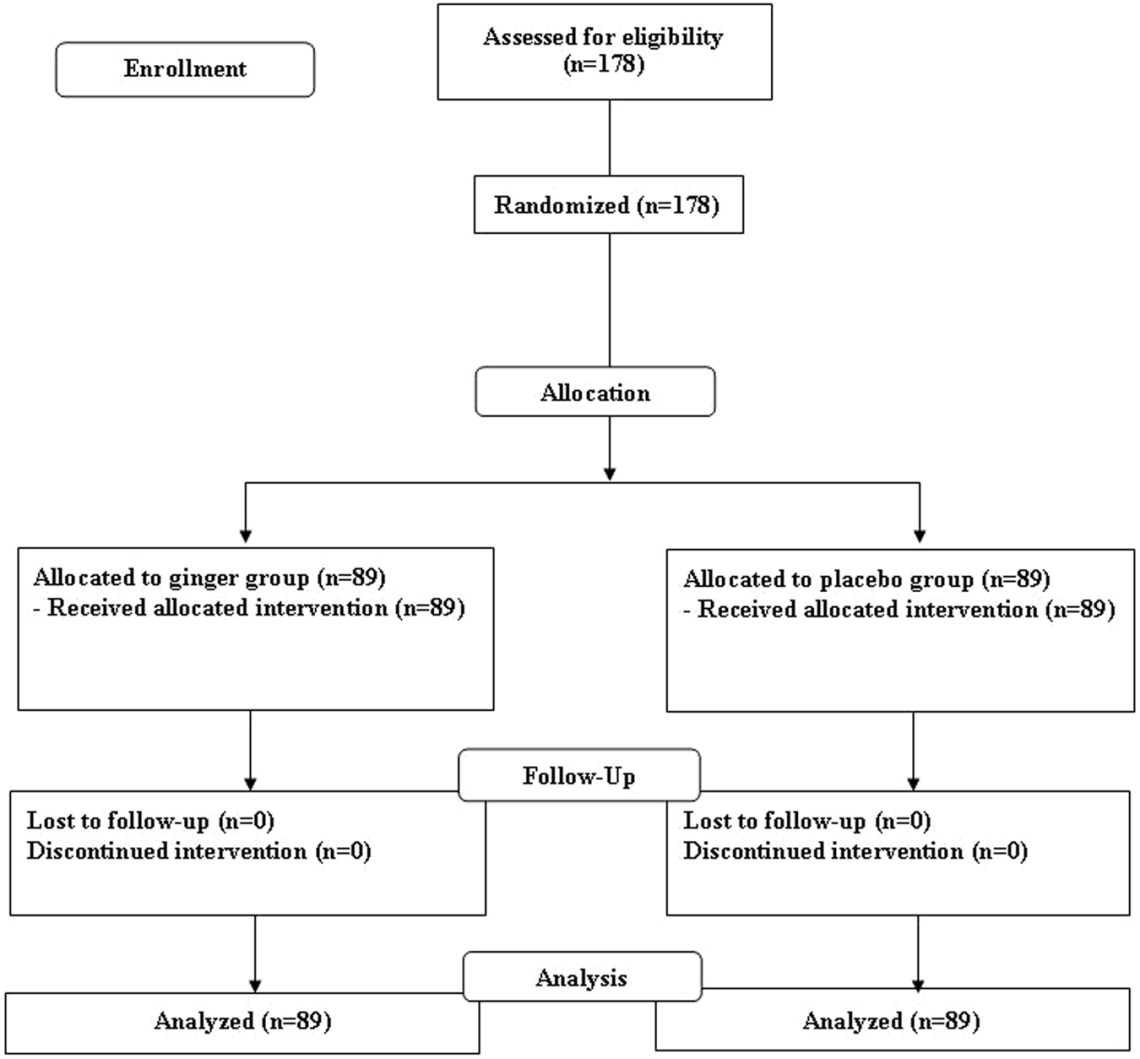
Patient follow-up profile after randomization to either ginger or placebo group.

One hundred and seventy-eight post cesarean section women were enrolled into the study (Fig. 1). All women were randomly assigned to two groups: 89 received ginger and 89 received placebo (Fig. 1). All women completed the study. For background characteristics, there were no significant differences between the groups with respect to maternal age, parity, gestational age at delivery, type of cesarean section, type of skin incision, operative time, history of abdominal discomfort before pregnancy, abdominal discomfort before intervention, compliance, and length of hospital stay (Table 1). Only mean estimated blood loss during operation was higher in the placebo group than the ginger group (464.3 ± 177.3 ml vs 414.0 ± 153.6 ml, p = 0.045).

**Table 1:** Baseline characteristics.

Baseline characteristics	Ginger group (n = 89)	Placebo group (n = 89)	P value
Maternal age	32.2 ± 4.9	33.5 ± 5.2	0.075
Multiparity	47 (52.8%)	48 (53.9%)	0.88
GA at delivery	38.2 ± 1.2	37.9 ± 1.0	0.062
Type of cesarean section			0.407
- Elective	61 (68.5%)	66 (74.2%)	
- Emergency	28 (31.5%)	23 (25.8%)	
Type of skin incision			0.11
- Low midline	40 (44.9%)	30 (33.7%)	
- Pfannenstiel	49 (55.1%)	59 (66.3%)	
Operative time (min)	38.3 ± 13.8	38.7 ± 13.0	0.841
Estimate blood loss (ml)	414.0 ± 153.6	464.3 ± 177.3	0.045
History of abdominal discomfort before pregnancy	31 (34.8%)	27 (30.3%)	0.522
Abdominal discomfort before intervention (VAS)	25 (5, 45)	20 (0, 50)	0.879
Good compliance	89 (100%)	89 (100%)	1.000
Length of hospital stay (days)	4.6 ± 1.2	4.5 ± 1.0	0.403

Data presented as mean ± SD, n (%) or median (interquartile range).

VAS: visual analogue scale.

The incidence of postoperative abdominal distention was not different between the ginger and placebo groups (20.2% vs 29.2%, p = 0.328). The severity of abdominal distention on the first – third day after operation was not different between groups, but on the fourth day after operation, the severity of abdominal distention was significantly lower in the ginger group than the placebo group (median visual analogue scale 10 vs 20, p = 0.036) (Table 2). The efficacy to relieve abdominal distention was also superior in the ginger group; more women in the ginger group felt that the drug can improve their symptom when compared to the placebo group (91% vs 65.2%, p < 0.001).

**Table 2:** Abdominal distention on the second day after operation, severity of abdominal distention on the first four days after operation, and drug efficacy.

	Ginger group (n = 89)	Placebo group (n = 89)	P value
Abdominal distention at the second day	18 (20.2%)	26 (29.2%)	0.328
Severity of abdominal distention (VAS)			
-First day	30 (1, 50)	30 (4, 50)	0.851
-Second day	30 (10, 50)	40 (5, 60)	0.517
-Third day	20 (2, 47.5)	30 (5, 50)	0.104
-Fourth day	10 (0, 30)	20 (4.5, 37.5)	0.036
Drug efficacy	81 (91%)	58 (65.2%)	<0.001
Another drug used	6 (6.7%)	5 (5.6%)	0.573

Data presented as n (%) or median (interquartile range).

VAS: visual analogue scale.

With regards to quality of life, the number of patients who had the ability to eat were higher in the ginger group than in the placebo group (59.6% vs 43.8%, p = 0.035), but this was not significantly different compared to other aspects of their life (i.e., sleeping, daily activity and emotion) (Table 3).

**Table 3:** Quality of life, time to defecate, time to flatus, maternal satisfaction, and side effects.

Result	Ginger group (n = 89)	Placebo group (n = 89)	P value
Quality of life			
- Eating	53 (59.6%)	39 (43.8%)	0.035
- Sleeping	54 (60.7%)	45 (50.6%)	0.174
- Daily activity	55 (61.8%)	42 (47.2%)	0.051
- Emotion	53 (59.6%)	42 (47.2%)	0.098
Time to flatus (since post operation) (min)	1962.6 ± 842.2	2129.3 ± 864.9	0.197
Time to defecation (since post operation) (min)	3091.0 ± 887.6	3445.5 ± 942.0	0.048
Maternal satisfaction	48 (53.9%)	37 (41.6%)	0.098
Side effect	30 (33.7%)	29 (32.6%)	0.614
- constipation	13	17	
- nausea/vomiting	2	2	
- diarrhea	6	3	
- heartburn	6	2	
- others	3	5	

Data presented as mean ± SD or n (%).

The ginger group had shorter mean first time to defecate than the placebo group (51.5 ± 14.8 hours vs 57.4 ± 15.7 hours, p = 0.048). There were no significant differences in time to first flatus, maternal satisfaction, and side effects (Table 3).

## Discussion

This randomized, double blinded, placebo controlled trial evaluated the efficacy of ginger in the prevention of abdominal distention in post cesarean section women. This study showed that the incidence of postoperative abdominal distention was not different between the ginger and the placebo groups, but the severity of abdominal distention on the fourth day after operation was significantly lower in the ginger group than the placebo group.

Ginger has anti-inflammatory, antioxidant, anti-emetic, analgesic, and antimicrobial activities. These effects can be mainly ascribed to 6-gingerol and 6-shogaol, which represent the major compounds in ginger rhizomes^16,17^. At preclinical level, 6-gingerol showed efficacy in rats against cisplatin-induced nausea and vomiting^17^. Thus, ginger has been used for reducing hyperemesis, postoperative nausea/vomiting, nausea/vomiting from chemotherapy, for pain relief, and for ailments of the digestive system^5,6^.

In the present study, we found that ginger relieved abdominal distention than the placebo. The number of patients who had the ability to eat was higher in the ginger group than in the placebo group. The ginger group also had shorter first time to defecate than the placebo group. A possible explanation for this is that the ginger increased muscular activity in the digestive tract^7–9,18^. Previous studies demonstrated that ginger can stimulate gastric emptying, relieve constipation and flatulence by increasing muscular activity in the digestive tract^7–9,18^.

The strength of this study was that it is the first randomized, double blinded, placebo controlled trial conducted to evaluate the efficacy of ginger in the prevention of abdominal distention in post cesarean section women. The limitations of this study were that it was a single center study and short form (SF)-36 was not used to evaluate the quality of life. Another limitation was that abdominal distention was evaluated by extracting the information from history taking and physical examination.

In conclusion, ginger does not decrease the incidence of post cesarean section abdominal distention. But, ginger is more effective than the placebo in relieving the severity of abdominal distention on the fourth day after operation and improved the participants’ ability to eat. Defecation was faster in the ginger group. Ginger is effective in relieving the severity of abdominal distention, inexpensive, easily accessible because it is our folk herbal medicine and has no serious side effect. Ginger may be used as an alternative method to relieve abdominal distention in post cesarean section women.
